# *Pediococcus pentosaceus* CECT 8330 protects DSS-induced colitis and regulates the intestinal microbiota and immune responses in mice

**DOI:** 10.1186/s12967-022-03235-8

**Published:** 2022-01-15

**Authors:** Fang Dong, Fangfei Xiao, Xiaolu Li, Youran Li, Xufei Wang, Guangjun Yu, Ting Zhang, Yizhong Wang

**Affiliations:** 1 Department of Gastroenterology, Hepatology and Nutrition, Shanghai Children’s Hospital, Shanghai Jiao Tong University, 355 Luding Road, Shanghai, 200062 China; 2 Institue of Pediatric Infection, Immunity and Critical Care Medicine, Shanghai Children’s Hospital, Shanghai Jiao Tong University School of Medicine, Shanghai, 200062 China

**Keywords:** *Pediococcus pentosaceus*, Probiotic, Colitis, Gut microbiota, IBD

## Abstract

**Background:**

Compelling evidences demonstrated that gut microbiota dysbiosis plays a critical role in the pathogenesis of inflammatory bowel diseases (IBD). Therapies for targeting the microbiota may provide alternative options for the treatment of IBD, such as probiotics. Here, we aimed to investigate the protective effect of a probiotic strain, *Pediococcus pentosaceus* (*P. pentosaceus*) CECT 8330, on dextran sulfate sodium (DSS)-induced colitis in mice.

**Methods:**

C57BL/6 mice were administered phosphate-buffered saline (PBS) or *P. pentosaceus* CECT 8330 (5 × 10^8^ CFU/day) once daily by gavage for 5 days prior to or 2 days after colitis induction by DSS. Weight, fecal conditions, colon length and histopathological changes were examined. ELISA and flow cytometry were applied to determine the cytokines and regulatory T cells (Treg) ratio. Western blot was used to examine the tight junction proteins (TJP) in colonic tissues. Fecal short-chain fatty acids (SCFAs) levels and microbiota composition were analyzed by targeted metabolomics and 16S rRNA gene sequencing, respectively. The Kyoto Encyclopedia of Genes and Genomes (KEGG) and Cluster of orthologous groups of proteins (COG) pathway analysis were used to predict the microbial functional profiles.

**Results:**

*P. pentosaceus* CECT 8330 treatment protected DSS-induced colitis in mice as evidenced by reducing the weight loss, disease activity index (DAI) score, histological damage, and colon length shortening. *P. pentosaceus* CECT 8330 decreased the serum levels of proinflammatory cytokines (TNF-α, IL-1β, and IL-6), and increased level of IL-10 in DSS treated mice. *P. pentosaceus* CECT 8330 upregulated the expression of ZO-1, Occludin and the ratio of Treg cells in colon tissue. *P. pentosaceus* CECT 8330 increased the fecal SCFAs level and relative abundances of several protective bacteria genera, including *norank_f_Muribaculaceae*, *Lactobacillus*, *Bifidobacterium*, and *Dubosiella*. Furthermore, the increased abundances of bacteria genera were positively correlated with IL-10 and SCFAs levels, and negatively associated with IL-6, IL-1β, and TNF-α, respectively. The KEGG and COG pathway analysis revealed that *P. pentosaceus* CECT 8330 could partially recover the metabolic pathways altered by DSS.

**Conclusions:**

*P. pentosaceus* CECT 8330 administration protects the DSS-induced colitis and modulates the gut microbial composition and function, immunological profiles, and the gut barrier function. Therefore, *P. pentosaceus* CECT 8330 may serve as a promising probiotic to ameliorate intestinal inflammation.

**Supplementary Information:**

The online version contains supplementary material available at 10.1186/s12967-022-03235-8.

## Background

Inflammatory bowel diseases (IBD) is a group of inflammatory disorders of the digestive tract that mainly characterized by chronic and relapsing inflammation of the gut mucosa [1]. IBD occurs in both children and adults, which most of the patients are diagnosed at young age [2]. Although IBD is usually considered as a common intestinal inflammatory disease of western countries, it has become a global disease for its rapidly increased prevalence and incidence in newly industrialized countries, including China [3, 4]. The initial clinical symptoms of IBD include diarrhea, abdominal pain, hematochezia, fistulae, and perianal lesions, which may also affect other organs with extraintestinal manifestations [5]. Patients with IBD usually suffered lifelong episodes of remission and relapse that significantly impair their quality of life. Current evidences indicate that multiple factors, including genetic, environmental, immunological and microbial factors, are involved in the pathogenesis of IBD, however, the precise etiology of IBD remains to be full elucidated [6]. It is assumed that IBD is occurring in genetically susceptible individuals with a dysregulated immune response towards gut microbiota, and under the influence of environmental factors [7].

The human gut microbiota is made up of trillions of microbial cells, including bacteria, viruses, and fungi, which is vital to human health. Gut microbial cells play critical roles in maintaining both local and systemic homeostasis through interacting with the human immune, endocrine and nervous systems [8]. For their high abundance (approximately 99.9% of the cell population), bacteria have been extensively studied as the first targets of gut microbiome in the past decade. Previous studies have demonstrated that IBD is strongly associated with a gut bacterial dysbiosis of reduced biodiversity and imbalance composition [9–14]. Compared with healthy controls, an increase in number of species belonging to phyla Actinobacteria and Proteobacteria, and a decrease in phyla Firmicutes and Bacteroidetes were observed in patients with IBD [15–17]. The abundances of bacteria with anti-inflammatory properties were decreased, while the relative abundances of pro-inflammatory bacteria were increased in patients with IBD [18]. Our previous studies revealed low relative abundances of short-chain fatty acids (SCFAs)-producing bacteria including *Faecalibacterium*, *Clostridium* clusters IV and XIVb, *Roseburia*, and *Ruminococcus* in pediatric Crohn’s disease (CD) patients [19].

Currently, there are only symptomatic treatments for IBD available, such as anti-inflammatory drugs (e.g., 5-Aminosalicylates), immunomodulatory agents (e.g., corticosteroids), and biologic treatments (e.g., tumor necrosis factor (TNF) inhibitors), which aim to reach clinical remission and mucosal healing [20]. However, the current therapies are limited by the variable efficacy, serious side effects and long-term safety. Since compelling evidences demonstrated that gut microbiota dysbiosis is involved in the pathogenesis of IBD, microbial-based and microbial-targeted therapies for restoring the gut microbial balance provide alternative options for the treatment of IBD [21]. Recently, a number of clinical trials have been performed to investigate the efficacy of microbiota-based therapies, including probiotics, prebiotics, synbiotics, and fecal microbiota transplantation (FMT) on IBD therapy [21, 22]. For example, probiotic strains, *E. coli* Nissle 1917, probiotic complex VSL#3 were effective to induce clinical remission in patients with mild or moderate ulcerative colitis (UC), and *Lactobacillus rhamnosus* GG (LGG) alone or combine with mesalazine prolonged clinical remission in UC patients [23, 24]. Thus, well-defined probiotics may have the potential to become an alternative therapy for IBD.

In the current study, we aimed to investigate the protective effect of a probiotic strain, *Pediococcus pentosaceus* (*P. pentosaceus*) CECT 8330 on intestinal inflammation in a DSS-induced colitis mice model. We further examined the effects of *P. pentosaceus* CECT 8330 on regulation of the cytokine levels, gut barrier function, SCFAs levels, and the gut microbiota composition and function changes.

## Materials and methods

### P. pentosaceus CECT 8330 preparation

*P. pentosaceus* CECT 8330 strain isolated from fresh stool of healthy children [25] was recovered from Dipro AB-8330 Drops (AB-Biotics. S/A, Barcelona, Spain) in Man Rogosa Sharpe (MRS, Sigma-Aldrich, USA) plate for 24 h in microaerophilic conditions (5% CO_2_) at 37 °C, as previously described [26]. Single colony was picked from the plate and further amplified anaerobically overnight at 37 °C in MRS broth. Bacteria were collected by centrifugation at 1500×*g* and 4 °C for 10 min, and resuspended in sterile phosphate-buffered saline (PBS) to a density of 2.5 × 10^9^ CFU/ml after washing with PBS for 3 times.

### Animals, probiotic treatment, and colitis induction

**Fig. 1:**
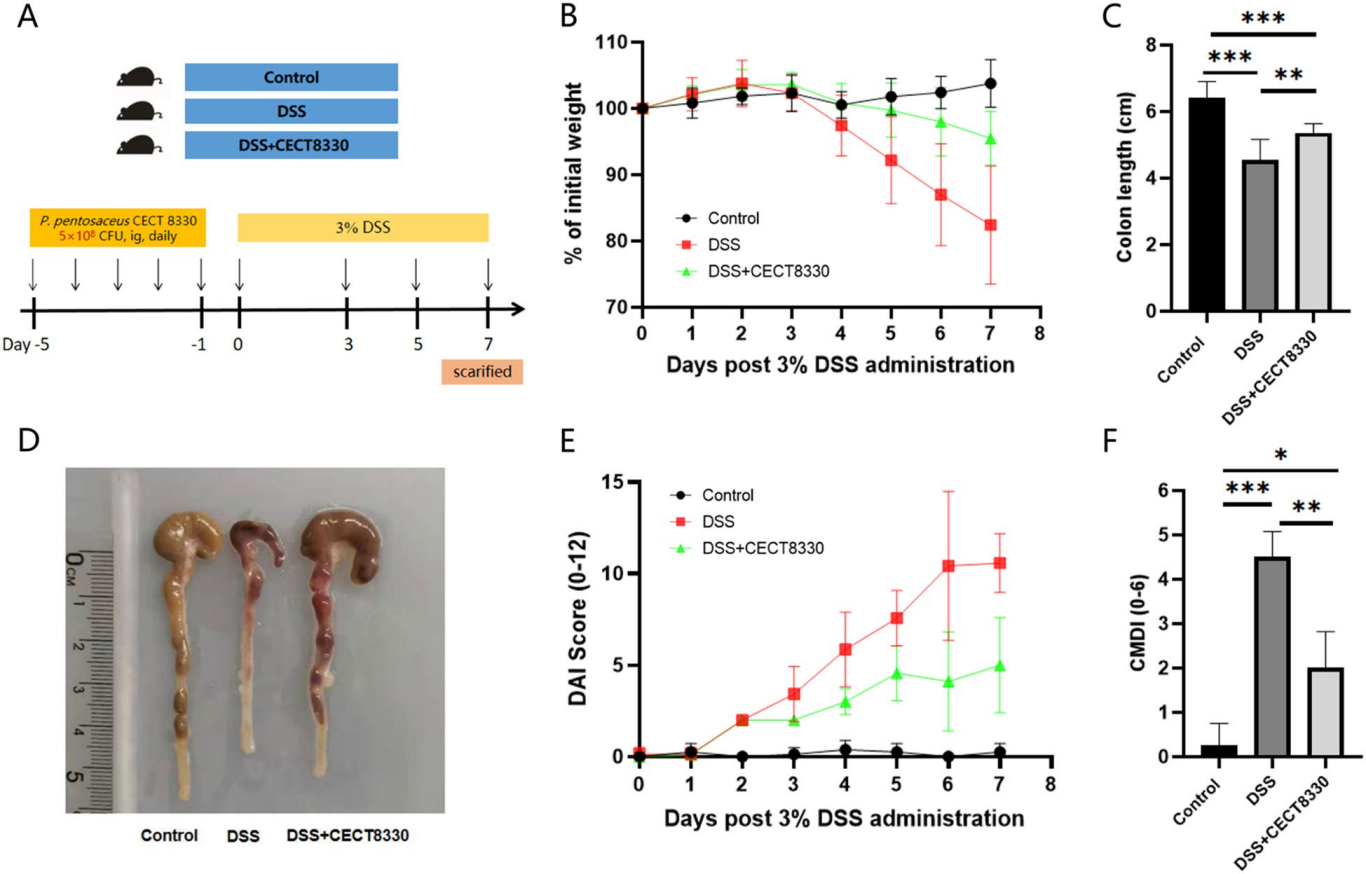
*P. pentosaceus* CECT 8330 protects DSS-induced colitis in female mice. **A** Schematic of animal experimental procedures (6 mice/group). **B** Changes of body weight (%). **C** Colon length shortening at day 7. **D** Representative images of the colon at day 7. **E** Disease activity index (DAI) scores. **F** Colon mucosal damage index (CMDI) scores at day 7. Significance was determined by ANOVA with Tukey’s analysis, *P < 0.05, **P < 0.01, ***P < 0.001

C57BL/6 mice (6 weeks old, 18–20 g) purchased from Ziyuan Lab (Zhejiang, China) were housed under standard specific pathogen-free (SPF) laboratory conditions (temperature, 20–24 °C; relative humidity, 50–60%; light cycle, 12/12 h light/dark). All animal experimental procedures in this study were approved by the Animal Ethics Committee of Shanghai Children’s Hospital (SHCH-IACUC-2020-XMSB-10). After one week of acclimation, mice were randomly divided into three groups: Control group, dextran sulfate sodium (DSS) group, and DSS + CECT 8330 group. All mice in the DSS + CECT 8330 group were treated with 200 μL *P. pentosaceus* CECT 8330 resuspension (5 × 10^8^ CFU) by oral gavage from day − 5 to − 1 prior to DSS administration. Mice in the control group and DSS group were given 200 μL sterile PBS by oral gavage daily instead of the probiotic (Fig. 1A). From day 0, mice in the DSS group and DSS + CECT 8330 group were given 3% (w/v) DSS (molecular weight: 36–50 kDa, MP Biomedicals, Santa Ana, CA, USA) ad libitum in drinking water for 7 days to induce colitis. Mice in the Control group were given sterile water during the study period. In the experiment including a posttreatment group, mice in the DSS + CECT 8330 (post) group were oral gavaged daily with 200 μL *P. pentosaceus* CECT 8330 resuspension (5 × 10^8^ CFU) from day 3 to 7 of DSS administration, and same volume of sterile PBS instead of the probiotic was given to the mice in other three group (Additional file 1: Fig. S1A).

### Disease activity index and colon mucosal damage index assessments

All mice were monitored daily for body weight and fecal conditions following the initiation of DSS treatment. A disease activity index (DAI) was recorded based on body weight loss (no loss: 0 point; 1–5% loss: 1 point; 5–10% loss: 2 points; 10–15% loss: 3 points; over 15% loss: 4 points), fecal consistency (normal: 0 point; loose stools: 2 points; watery diarrhea: 4 points), and hematochezia (no bleeding: 0 point; slight bleeding: 2 points; gross bleeding: 4 points). The DAI score (0–12) was the total score of above three parameters [27]. The colonic mucosa damage index (CMDI) was assessed at the time of killing on day 7 by ulceration and inflammation (normal: 0 point; focal hyperemia, no ulcers: 1 point; ulceration without hyperemia or bowel wall thickening: 2 points; ulceration with inflammation at one site: 3 points; two sites of ulceration and inflammation: 4 points; major sites of damage extending ≥ 1 cm along the colon: 5 points; damage extending ≥ 2 cm along the length of the colon, with the score increasing by 1 point for each additional cm of damage: 6–10 points), and presence of adhesions (normal: 0 point; minor adhesions: 1 point; major adhesions: 2 points) [28].

### Histological analysis

A 5 mm long distal colon sample of each mouse was collected and fixed in 4% paraformaldehyde fixative solution for 24 h, then sample was embedded in paraffin wax. Sample was sliced into 5 µm sections for hematoxylin and eosin (H&E) staining. Randomly selected slices from each group were observed with a pathologic slice scanner at 100 × magnification, and then recorded photomicrographs. A histological index (HI) calculating based on inflammatory cell infiltration (1–3 points) and the intestinal architecture (1–3 points) was used to assess the degree of the histopathological changes, as previously described [29].

### Western blot analysis

Approximately 50 mg of colon tissue was frozen in liquid nitrogen and pulverized. Total protein was extracted by using 1 × sodium dodecyl sulfate (SDS) lysis buffer with 1% phenylmethylsulfonyl fluoride (PMSF) for 30 min at 4 °C, and the supernatant was collected after centrifugation (12,000 rpm, 5 min). The protein concentration was quantified using the BCA kit. Equal amount of protein (50 μg) was separated by 8% and 10% SDS‐polyacrylamide gel electrophoresis (SDS‐PAGE) for 2 h and transferred to polyvinylidene difluoride (PVDF) membrane. The membrane was blocked with 5% nonfat milk for 2 h at room temperature and incubated with antibodies specific for GAPDH (Cell Signaling Technology; #5714; 1:1000), β-Actin (Cell Signaling Technology; #8457; 1:1000), ZO-1 (Cell Signaling Technology; #8193; 1:1000), and Occludin (Bioworld; BS72035; 1:1000) at 4 °C overnight. Subsequently, the membrane was incubated for 1 h with secondary antibody (Cell Signaling Technology; #7074; 1:1000), and detected by Bio-Rad Chemi Doc XRS plus an image analyzer (Bio-Rad, Hercules, CA, USA).

### Serum cytokine analysis

At the time of sacrifice on day 7, mice were anesthetized with chloral hydrate (2.5%, 0.1 mL/10 g) and removed the eyeballs to collect the blood samples. The serum was obtained by centrifugation at 3000 rpm for 10 min. Cytokines, interleukin-6 (IL-6), IL-10, IL-1β, and TNF-α in the serum were determined using ELISA kits (4A Biotech Co. Ltd., Beijing, China) following the manufacturer’s instructions, respectively.

### Flow cytometry analysis

The colon tissue selected from 0.5 cm below the cecum to 0.5 cm above the anus was cut longitudinally and then transversely into 0.5 cm pieces after removing adipose tissue, mesenteric connective tissue, and Peyer's patches. After washing with PBS, colon pieces were digested with collagenase solution containing 1 mg/mL collagenase VIII and 1 U/mL DNase I (Gibco, Life Technologies) for 55 min in a 37 °C shaker. The supernatant was filtered with 40 μm cell strainer, and centrifuged at 2000 rpm for 5 min. The cell suspension was centrifuged after washing with plain RPMI 1640. Lamina propria lymphocytes (LPLs) were separated by density gradient centrifugation (cells were resuspended with 40% Percoll solution, and overlaid with an 80% Percoll solution, at 2500 rpm, 25 min). The interface containing the LPLs were aspirated and washed in medium, and subjected to stain with anti-CD4 (clone GK1.5; eBioscience), anti-CD25 (clone PC61; eBioscience) at 4 °C in the dark for 30 min. Cells were fixed and permeabilized with 200 μL fixation-permeabilization buffer overnight at 4 °C in the dark. Subsequently, cells were incubated intracellularly with Foxp3-PE (clone NRRF-30; eBioscience) at 4 °C in the dark for 1 h, and were analyzed by flow cytometry (Cyto Flex S). The data were analyzed by FlowJo software (Tree Star).

### SCFAs analysis

Fecal levels of SCFAs were measured by gas chromatography-mass spectrometry (GC–MS) as previously described [30]. Briefly, 100 mg fecal sample was accurately weighed, and the SCFAs were extracted using a 1000 µL aliquot of methanol containing 100 µL internal standards. After grinding the mixture and sonication, the sample was settled at − 20 °C for 30 min. Then, the sample was centrifuged at 13,000×*g* at 4 °C for 15 min. A 200 µL of supernatant was transferred to a new 1.5 mL tube. Finally, 50 mg anhydrous sodium sulfate was added to the tube and vortex, after centrifugation (13,000×*g*, 4 °C, 15 min), the supernatant was carefully transferred to sample vial for further analysis using Agilent 8890B-5977B GC/MS system and HP-INNOWAX (30 m × 0.25 mm × 0.25 µm) capillary column.

### Fecal microbiome analysis

Total genomic DNA was extracted from fecal samples using QIAamp DNA Stool Mini Kit (Qiagen, Germany) according to manufacturer’s instructions. Isolated genomic DNA was amplified for the 16S rRNA V3-V4 hypervariable regions using primer pairs 338F (5′-ACTCCTACGGGAGGCAGCAG-3′) and 806R (5′-GGACTACHVGGGTWTCTAAT-3′). The PCR products were purified with AxyPrep DNA Gel Extraction Kit (Axygen Biosciences, CA, USA). Sequencing libraries were generated using the NEXTFLEX® Rapid DNA-Seq Kit. Sequencing was performed using Illumina MiSeq platform (Illumina, San Diego, USA) according to the standard protocol (Majorbio Bio-Pharm Technology Co. Ltd., Shanghai, China). The raw 16S rRNA gene sequences were quality-filtered by fastp (version 0.20.0) and merged by FLASH (version 1.2.7). Operational taxonomic units (OTUs) were assigned a 97% similarity cutoff for clustering (UPARSE version 7.1) and chimeric sequences were identified and removed. OTUs were analyzed by RDP Classifier version 2.2 against the 16S rRNA database (eg. Silva v138) using confidence threshold of 0.7. The *Alpha* diversity was measured using the Chao, Simpson, Ace and Observed species indexes. The *beta* diversity was conducted through Principal coordinate analysis (PCoA) according to Bray–Curtis distance. The abundance of taxa difference between groups was analyzed using Kruskal–Wallis test. Differential enrichment of gut microbiota was analyzed by linear discriminant analysis (LDA) effect size (LEfSe). Functional profiles of pathway enrichment analysis was performed using Kyoto Encyclopedia of Genes and Genomes (KEGG) and cluster of orthologous groups of proteins (COG) database by Phylogenetic Investigation of Communities by Reconstruction of Unobserved States (PICRUSt) [31]. Spearman correlation was applied to investigate the associations between fecal SCFAs, serum cytokines, and the composition of the gut microbiome.

### Statistical analysis

Data were presented as mean ± SEM or median with interquartile range. To determine significance among the three groups, the Kruskal–Wallis test was used for non-normally distributed data analysis, and one-way ANOVA followed by Tukey’s test was used for normally distributed data. All statistical analyses and graph generation were performed by using Graph Pad Prism (version 9.0.0). P < 0.05 was considered to be statistically significant.

## Results

### *P. pentosaceus* CECT 8330 protects DSS-induced colitis

**Fig. 2:**
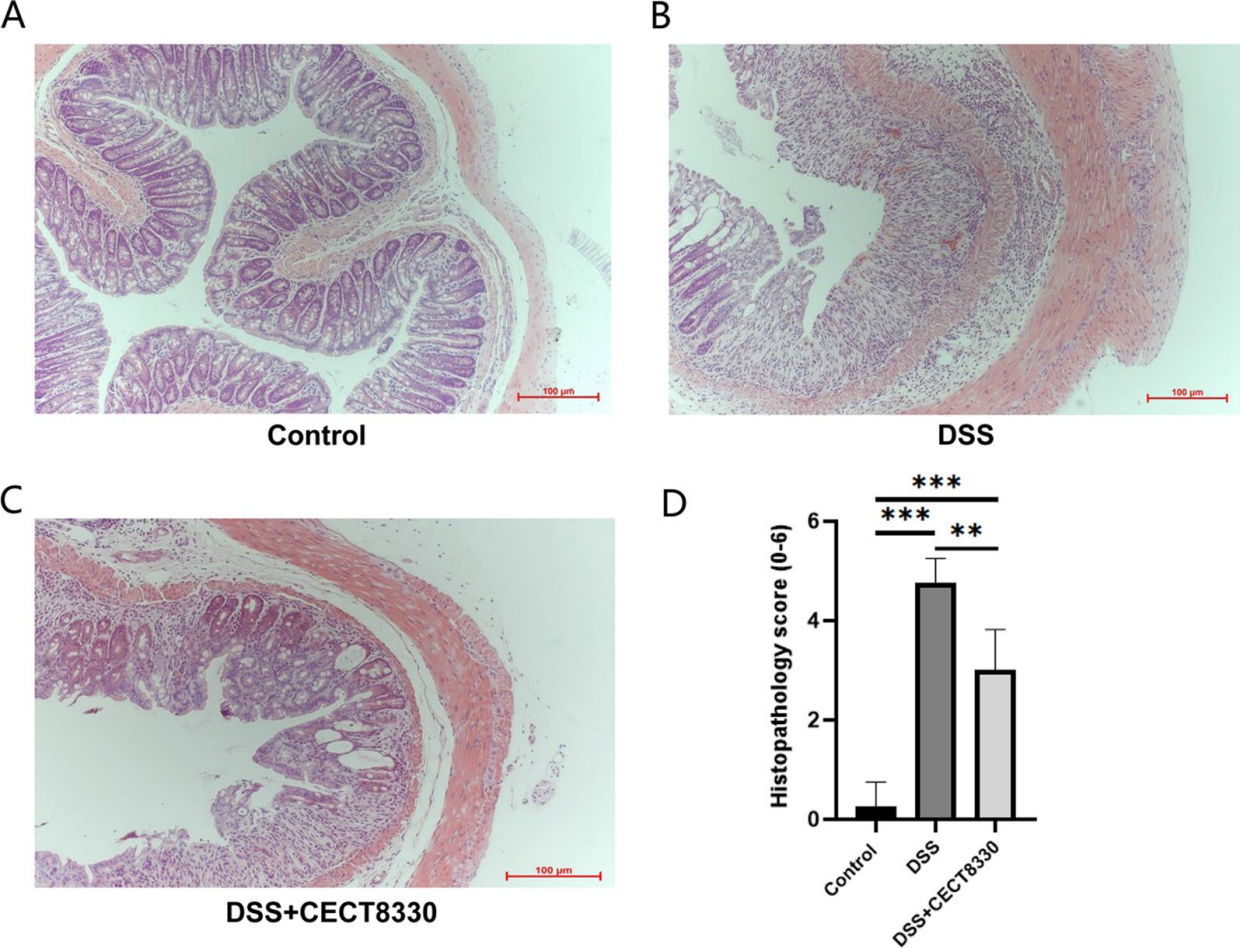
*P. pentosaceus* CECT 8330 protects DSS-induced colon epithelial damage. Representative H&E- stained colon sections (magnification 100 ×) images (**A**, **B**, **C**) and histopathology score (**D**) in Control, DSS and DSS + CECT 8330 groups. Significance was determined by ANOVA with Tukey’s analysis, **P < 0.01, ***P < 0.001

To examine the protective effect of *P. pentosaceus* CECT 8330 on DSS-induced colitis in mice, we treated the mice with *P. pentosaceus* CECT 8330 prior to or 2 days after DSS administration for 5 days (Fig. 1A, Additional file 1: Fig. S1A). As shown in Fig. 1, *P. pentosaceus* CECT 8330 treatment significantly protected DSS-induced loss of weight (Fig. 1B, Additional file 1: Fig. S1B), shortening of colon length (Fig. 1C, D, Additional file 1: Fig. S1C, D), DAI (Fig. 1E, Additional file 1: Fig. S1E) and CDMI (Fig. 1F, Additional file 1: Fig. S1F) scores in both female and male mice. Histologically, control groups showed intact colonic epithelial cells (Fig. 2A, Additional file 1: Fig. S2A), while DSS group showed incomplete mucosal structures, crypt abscesses, ulcers, and extensive inflammatory cell infiltration in the colonic tissues (Fig. 2B, Additional file 2: Figure S2B). *P. pentosaceus* CECT 8330 partially protected the mucosal architecture and goblet cell loss, and reduced inflammatory cell infiltration (Fig. 2C, Additional file 2: Figure S2C). The HI score was significantly decreased in mice treated with *P. pentosaceus* CECT 8330 (Fig. 2D, Additional file 2: Figure S2E). In addition, administration of *P. pentosaceus* CECT 8330 from day 2 to 7 of DSS treatment also protected the symptoms of colitis (Additional file 1: Figure S1, Additional file 2: Figure S2D and E).

### *P. pentosaceus* CECT 8330 induces tight junction proteins expression

**Fig. 3:**
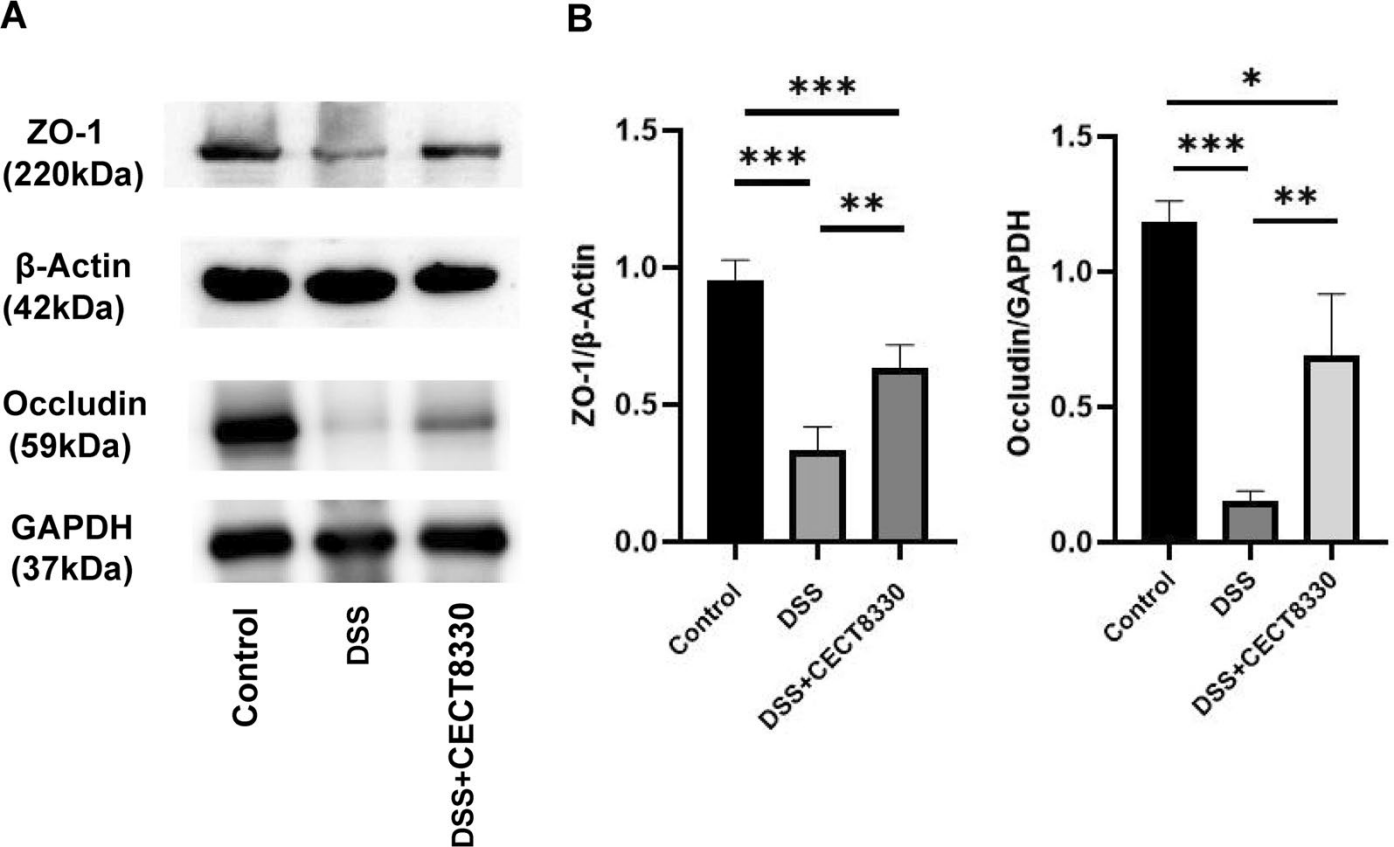
*P. pentosaceus* CECT 8330 increases tight junction proteins expression. **A** Representative experiment of ZO-1 and Occludin proteins in the colon examined by Western blot. **B** The relative amounts of ZO-1 and Occludin calculated by densitometry of protein bands from three independent experiments. β-Actin and GAPDH were used as loading controls, respectively. Significance was determined by ANOVA with Tukey’s analysis, *P < 0.05, **P < 0.01, ***P < 0.001

The intestinal epithelial cells are linked by tight junctions, which form the intestinal barrier. Tight junctions are composed of transmembrane and cytoplasmic scaffolding tight junction proteins (TJP), including ZO-1 and occludin. Different kinds of TJP interact with each other tightly to form an integrated intestinal epithelial barrier [32]. Thus, we next examined ZO-1 and Occludin expression in colon tissue using Western blot. As presented in Fig. 3, colon tissue of mice from DSS + CECT 8330 group showed increased ZO-1 and Occludin protein levels as compared with DSS group.

### *P. pentosaceus* CECT 8330 increases the ratio of CD4^+^CD25^+^Foxp3^+^ Treg cells

**Fig. 4:**
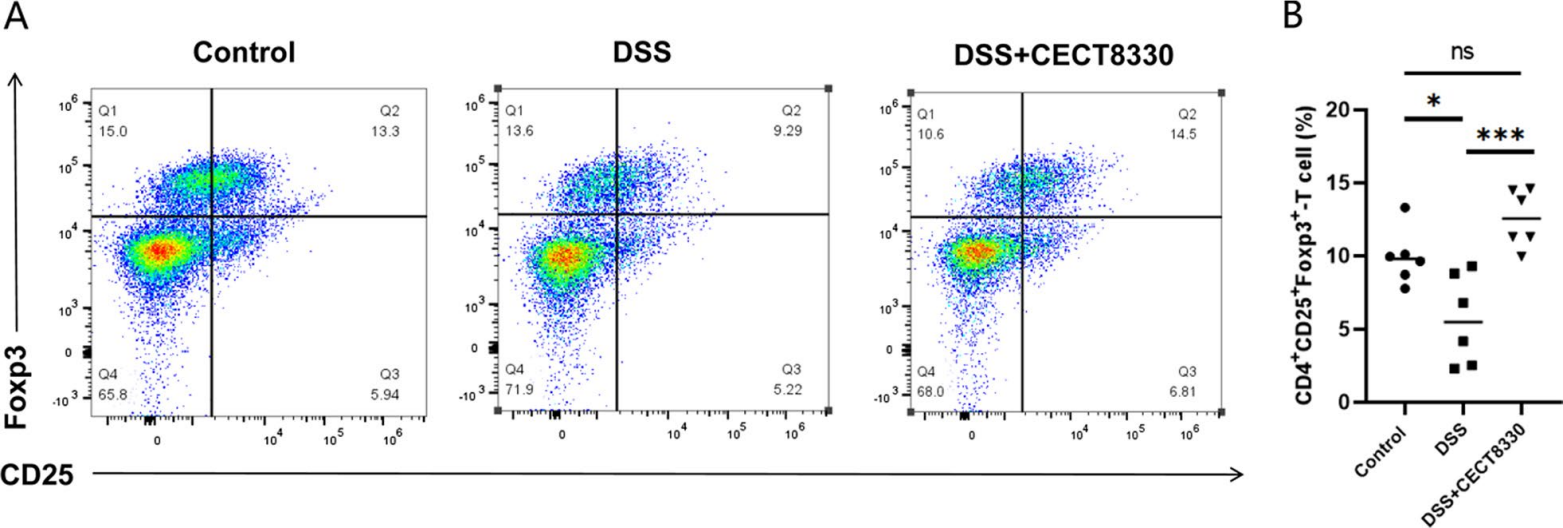
*P. pentosaceus* CECT 8330 increases the ratio of CD4^+^CD25^+^Foxp3^+^ Treg cells in the colon. **A** Representative experiment of the ratio of CD4^+^CD25^+^Foxp3^+^ Treg cells determined by flow cytometry. **B** Bar charts showing the percentage of Treg cells in colon samples of Control, DSS and DSS + CECT 8330 groups. Significance was determined by ANOVA with Tukey’s analysis. *P < 0.05, ***P < 0.001

We examined the ratio of CD4^+^CD25^+^FOXP3^+^ Treg cells in colonic LPLs of mice from three groups using flow cytometry. As presented in Fig. 4, the proportion of CD4^+^CD25^+^FOXP3^+^ Treg cells was significantly downregulated in DSS group, while *P. pentosaceus* CECT 8330 treatment significantly increased the ratio of CD4^+^CD25^+^FOXP3^+^ Treg cells in the colonic tissue.

### *P. pentosaceu* CECT 8330 modulates cytokine expression

**Fig. 5:**
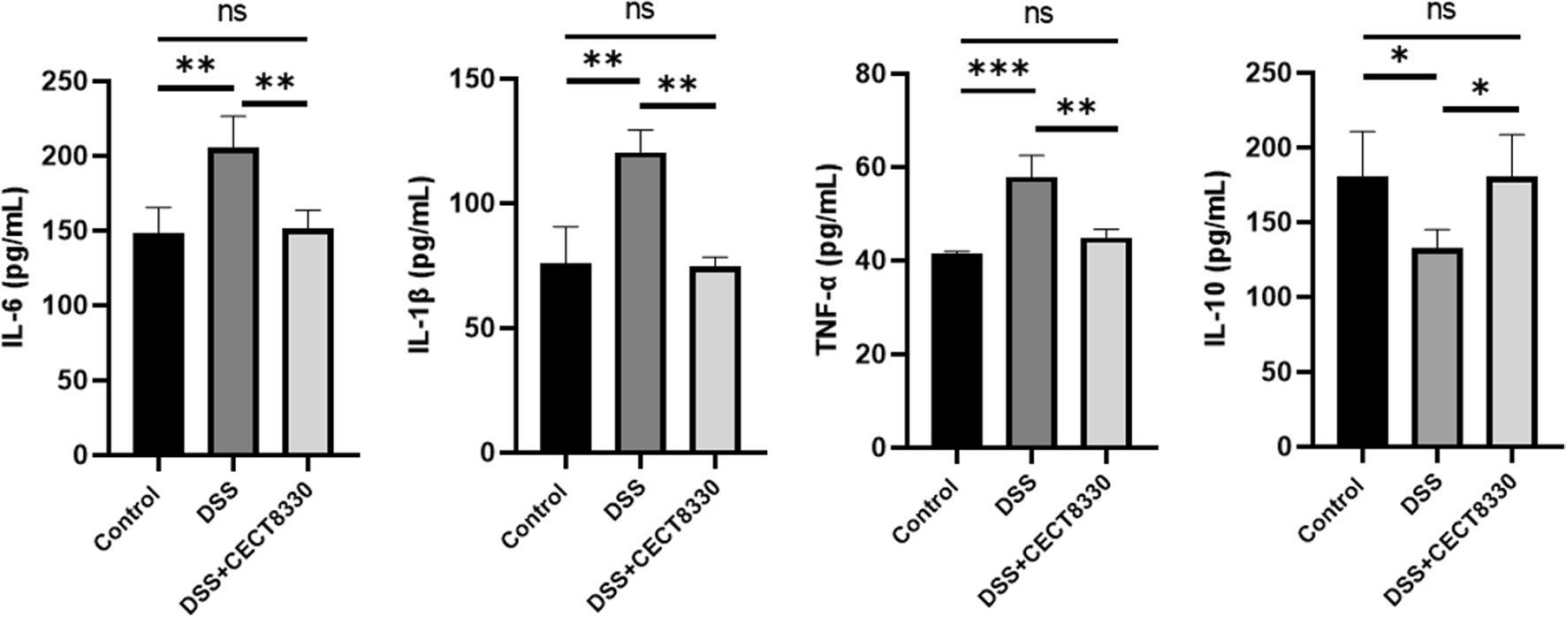
*P. pentosaceus* CECT 8330 modulates serum cytokines levels in DSS treated mice. Serum IL-6, IL-1β, TNF-α, and IL-10 levels were measured by ELISA in mice from the Control, DSS and DSS + CECT 8330 groups. Significance was determined by ANOVA with Tukey’s analysis. *P < 0.05, **P < 0.01, ***P < 0.001

As shown in Fig. 5, serum levels of the inflammatory cytokines, IL-1β, TNF-α, IL-6 were significantly increased, and IL-10 level was decreased in mice from DSS group as compared with Control group. *P. pentosaceu* CECT 8330 treatment decreased the serum levels of IL-1β, TNF-α, IL-6, and increased the IL-10 level.

### *P. pentosaceus* CECT 8330 modulates the fecal SCFAs levels

**Fig. 6:**
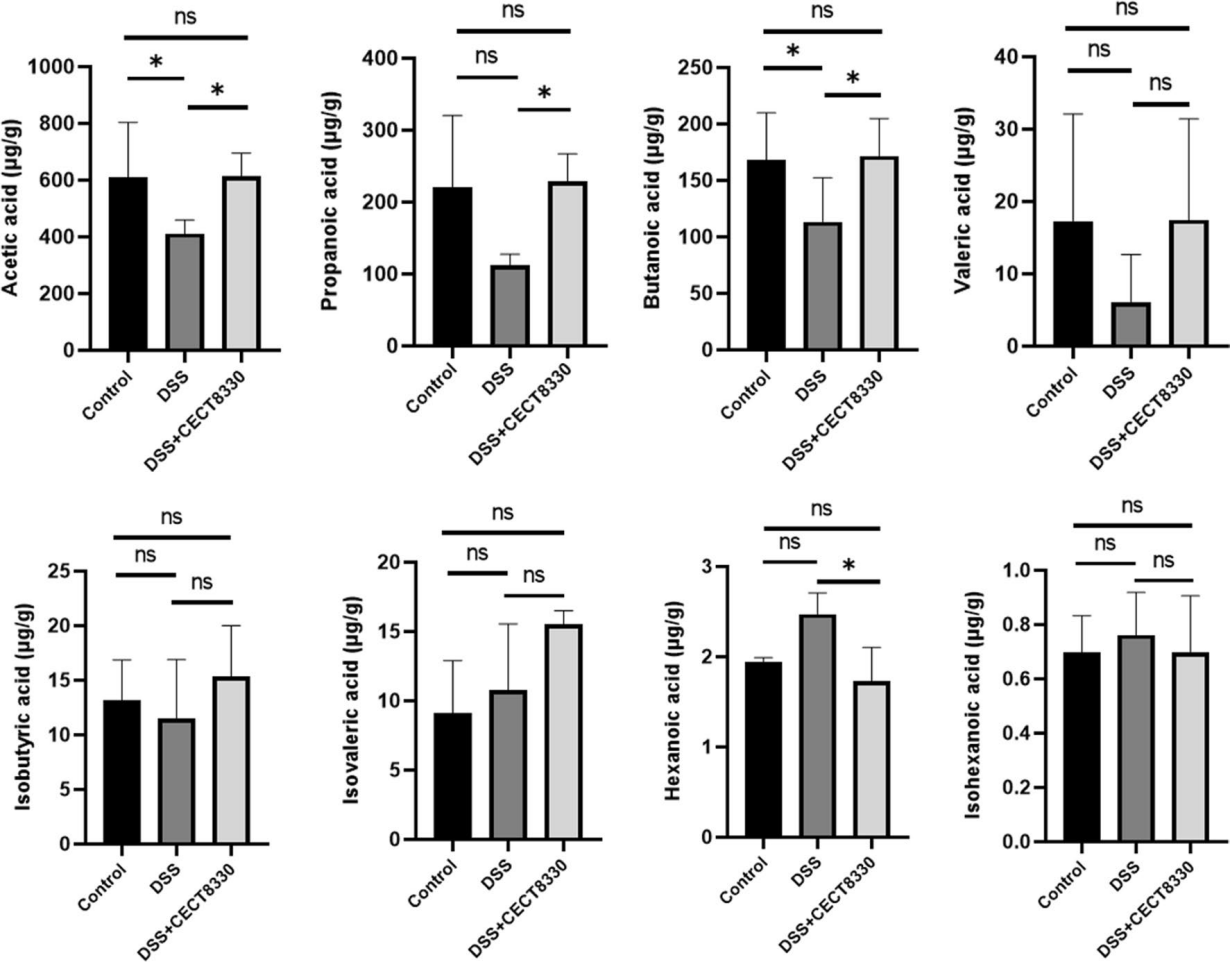
*P. pentosaceus* CECT 8330 increases the levels of short-chain fatty acids (SCFAs). Acetic acid, propionic acid, butyric acid, valeric acid, isobutyric acid, isovaleric acid, hexanoic acid, and ishexanoic acid of fecal samples from the Control, DSS and DSS + CECT 8330 groups were determined by using GC–MS. Significance was determined by ANOVA with Tukey’s analysis. *P < 0.05, **P < 0.01, ***P < 0.001

Compared with Control group, acetic acid, propionic acid, butyric acid, and valeric acid levels were decreased in DSS group. In contrast, *P. pentosaceus* CECT 8330 treatment increased the levels of acetic acid, propionic acid, butyric acid, and valeric acid. In addition, hexanoic acid level was elevated in DSS group, but *P. pentosaceus* CECT 8330 reduced the hexanoic acid level in DSS + CECT 8330 group. No significant changes were found in the levels of isobutyric acid, isovaleric acid, and isocaproic acid among the three groups (Fig. 6).

### *P. pentosaceus* CECT 8330 modifies gut microbiota composition

**Fig. 7:**
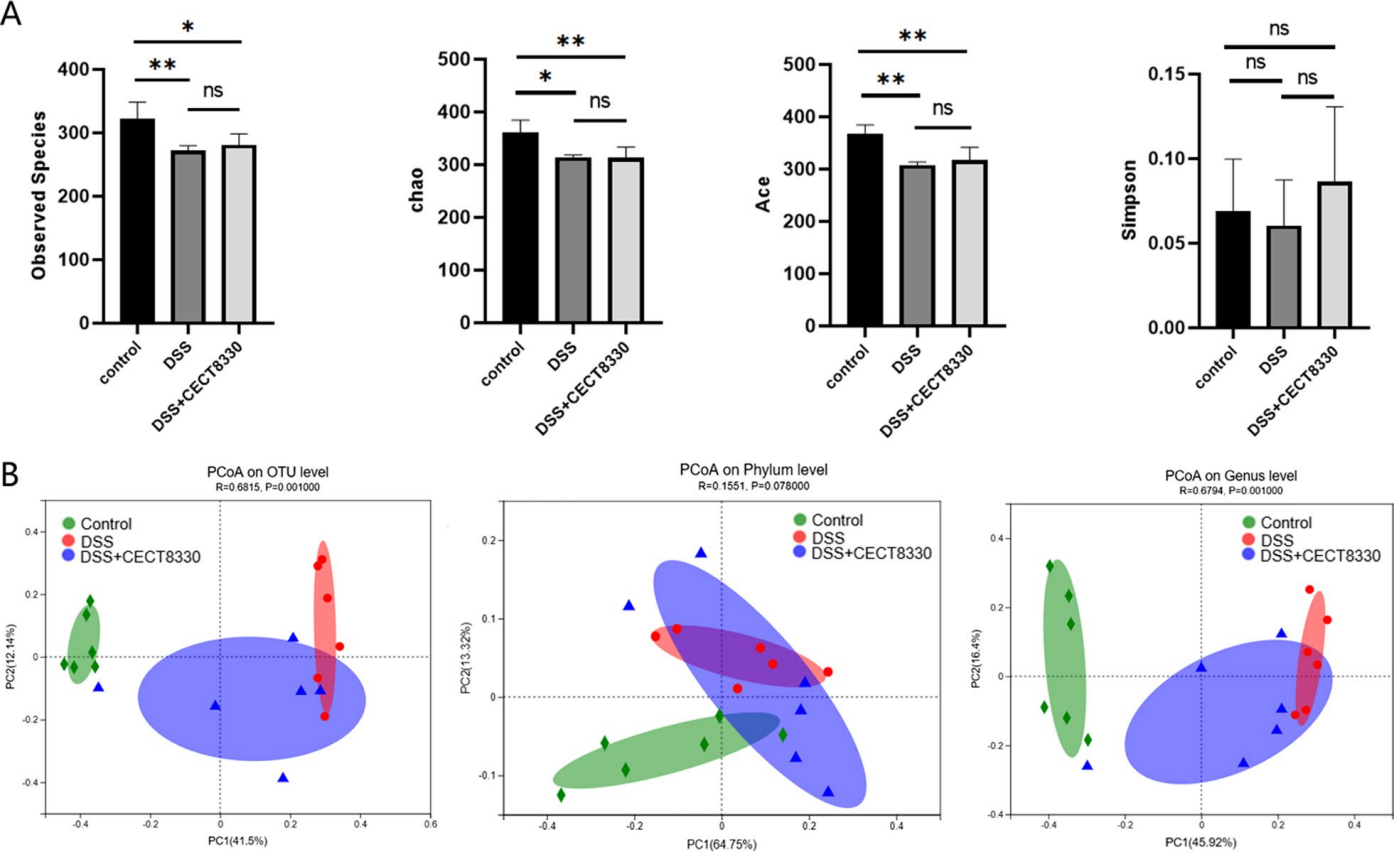
*P. pentosaceus* CECT 8330 modulates the gut microbiota diversity. **A** *Alpha* diversity index (observed species, Chao, Ace and Simpson) of the fecal microbiome in the Control, DSS and DSS + CECT 8330 groups at the OTU level. **B** *Beta* diversity of bacterial using Principal Coordinate analysis (PCoA) based on Bray–Curtis distance between the three groups at the phylum, genus, and OTU level. Significance was determined by Kruskal–Wallis test or ANOVA with Tukey’s analysis. *P < 0.05, **P < 0.01

Gut microbiota composition was determined by 16S rRNA gene high throughout sequencing. As shown in Fig. 7, analysis of *alpha* diversity revealed both the richness and diversity (calculated in observed species, ACE, Chao1, and Simpson indexes) were lower in DSS group and DSS + CECT 8330 group than Control group. Although an increasing trend of *alpha* diversity was observed in DSS + CECT 8330 group, no statistical difference was achieved as compared with DSS group. Analysis of the *beta* diversity calculated on the Bray–Curtis dissimilarity of observed OTUs, phylum level, genus level, showed that fecal microbial community of DSS group apart from that of Control group. *P. pentosaceus* CECT 8330 treatment modulated the gut microbiota composition of DSS-induced dysbiosis toward to Control group.

**Fig. 8:**
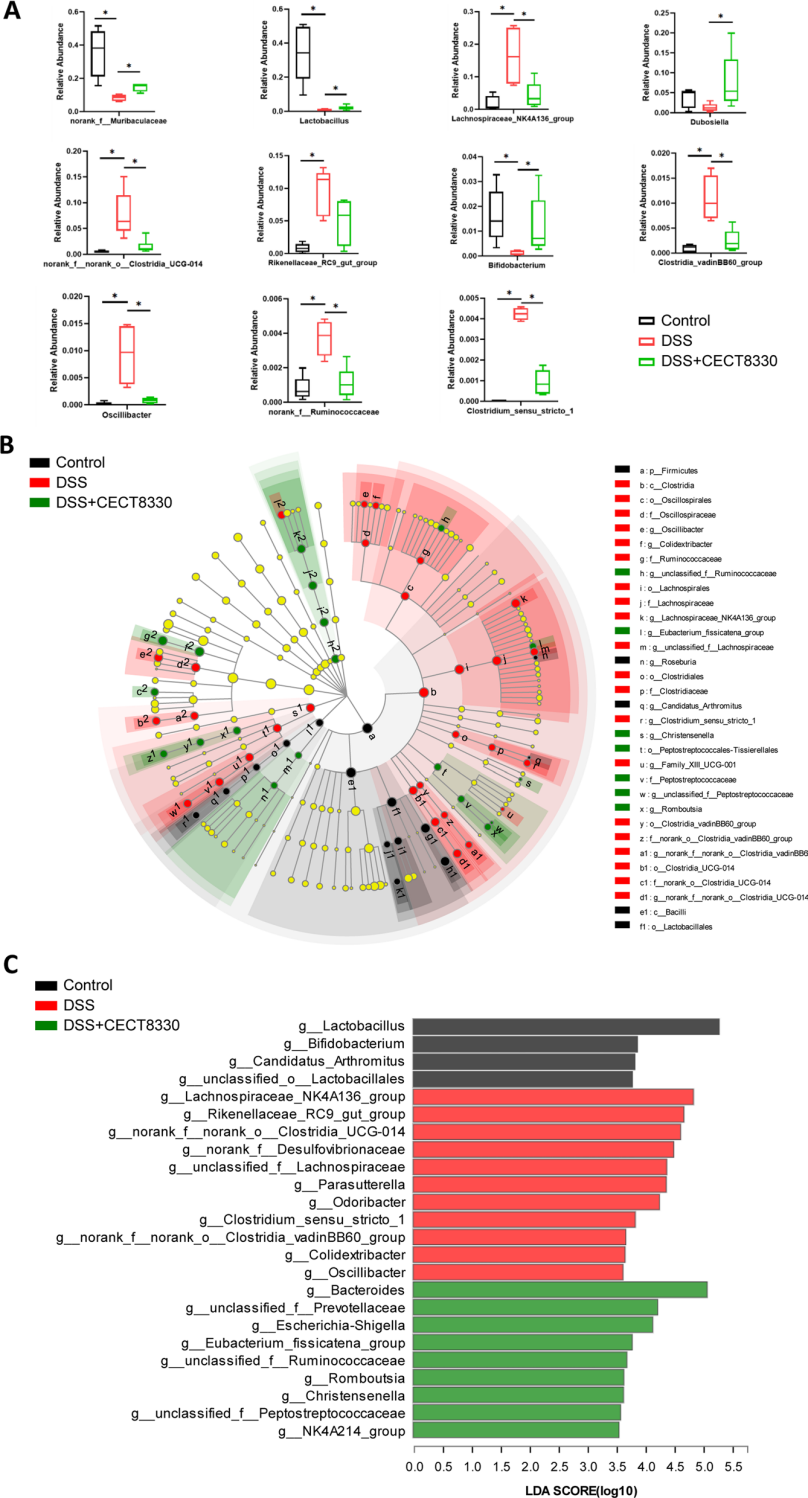
*P. pentosaceus* CECT 8330 modulates the abundance of bacteria genera and enrichment of gut microbiota. **A** Boxplots showing the 11 significantly different bacterial genera among the Control, DSS and DSS + CECT 8330 groups. Significance was determined by Kruskal–Wallis test or ANOVA with Tukey’s analysis. *P < 0.05. **B** Cladogram generated from LEfSe analysis showing the relationship between taxon (from the inner ring to the outer ring, the grades represent the phylum, class, order, family, genus and species) in the Control, DSS and DSS + CECT 8330 groups. Each dot represents a taxonomic hierarchy. **C** LEfSe analysis selected bacterial features associated with the Control, DSS and DSS + CECT 8330 groups. Genera with a linear discriminant analysis (LDA) score > 3.5 were plotted

The bacterial microbiota was dominated by phyla Firmicutes, Bacteroidetes, Proteobacteria, Tenericutes and Actinobacteria in all three groups (Additional file 3: Fig. S3). The top three most abundant genera in Control group were *norank_f_Muribaculaceae*, *Lactobacillus*, and *Lachnospiraceae_NK4A136_group* (Additional file 4: Fig. S4). Inter-group comparisons of taxonomic profiles at the genus level revealed that DSS group exhibited lower relative abundances of *norank_f_Muribaculaceae*, *Lactobacillus Bifidobacterium*, and *Dubosiella*, and higher relative abundances of *Lachnospiraceae_NK4A136_group*, *norank_f_norank_o_Clostridia_UCG-014*, *Clostridium_sensu_stricto_1*, *Clostridia_vadinBB60_group*, *Oscillibacter*, *GCA_900066575*, and *norank_f_Ruminococcaceae* as compared to Control group (Fig. 8A). Notably, supplementation with *P. pentosaceus* CECT 8330 clearly ameliorated this effect of DSS on the gut microbiota (Fig. 8A)*.*

LEfSe analysis identified 32 taxa that were differentially abundant in the Control, DSS and DSS + CECT 8330 groups. Phyla Firmicutes, class Bacilli, order Lactobacillales, and genera *Roseburia*, *Candidatus_Arthromitus* were enriched in Control group. In comparison with the Control group, DSS treatment increased the abundances of class Clostridai, 5 orders, including Oscillospirales and Clostridiaceae, 6 families (Oscillospiraceae, Ruminococcaceae, etc.), and 8 genera, such as *Colidextribacter, Oscillibacter*, while order Peptostreptococcales-Tissierellales, family Peptostreptoccoccaceae, and genera *unclassified_f_Ruminococcaceae, Eubacterium_fissicatena_group, Romboutsia, Christensenella,* and *unclassified_f_Peptostreptococcaceae* were enriched in DSS + CECT 8330 group (Fig. 8B). Furthermore, LDA scores (> 3.5) derived from LEfSe analysis at genus level identified several bacterial genera that discriminated the Control, DSS and DSS + CECT 8330 group. Notable high abundances of *Lactobaillus*, *Bifidobacterium*, *Candidatus_Arthromitus* were found in mice from the Control group, while the DSS enriched the abundances of *Lachnospiraceae_NK4A136_group*, *Rikenellaceae_RC9_gut_group*, *norank_f_norank_o_Clostridia_UCG-014*, *norank_f_Desulfovibrionaceae*, *unclassified_f_Lachnospiraceae*, *Parasutterella*, *Odoribacter*, *Clostridium_sensu_stricto_1*, *Clostridia_vadinBB60_group*, *Colidextribacter*, *Oscillibacter*, and *Oscillibacter*. The abundances of *unclassified_f_Prevotellaceae*, *Escherichai-Shigella*, *Eubacterium_fissicatena_group*, *unclassified_f_Ruminococcaceae*, *Romboutsia, Christensenella*, *unclassified_f_Peptostreptococcaceae*, and *NK4A214_group* were enriched in DSS + CECT 8330 group (Fig. 8C).

### Functional profile of the gut microbiome

**Fig. 9:**
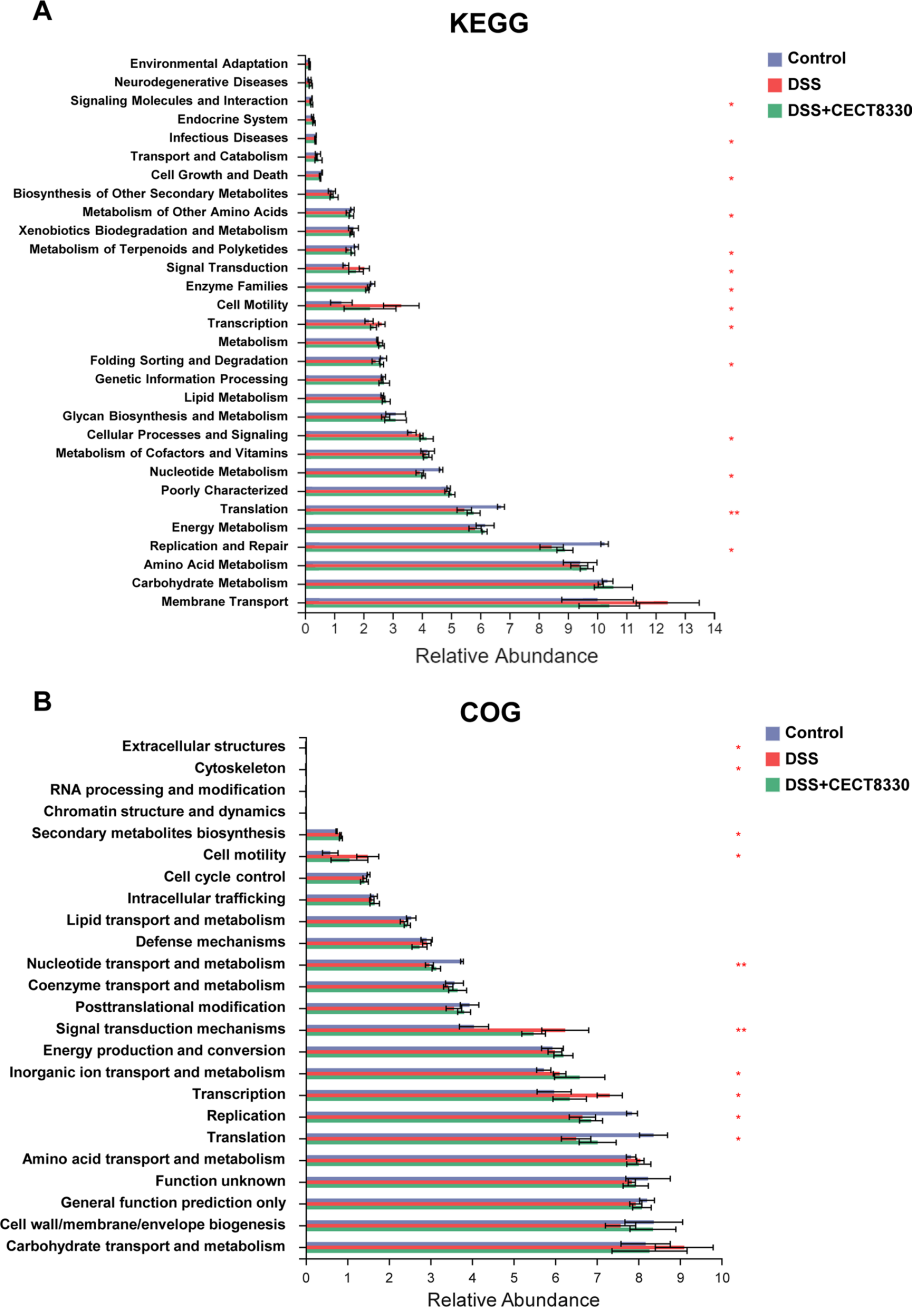
Functional profile of the gut microbiome. The bar charts of KEGG (**A**) and COG (**B**) pathways showing 14 and 10 significant differences among the Control, DSS and DSS + CECT 8330 groups, respectively. Significance was determined by Kruskal–Wallis test. *P < 0.05, **P < 0.01

KEGG and COG pathway analysis were performed to explore potential differences in the functional composition of the microbiome in three groups using PICRUSt. The microbiome of mice in DSS group showed lower abundance in KEGG pathways of replication and repair, translation, nucleotide metabolism, glycan biosynthesis and metabolism, folding, enzyme families, metabolism of terpenoids and polyketides, metabolism of other amino acids, cell growth and death, infectious diseases, signaling molecules and interaction than the mice in Control group (Fig. 9A). Higher abundance in KEGG pathways of cellular processes and signaling, cell motility, transcription, signal transduction was identified in DSS group (Fig. 9A). In addition, COG pathway analysis showed similar patterns of abundance changes, such as lower abundance of translation, ribosomal structure and biogenesis, and higher abundance of cell motility, transcription, and signal transduction mechanisms in DSS group (Fig. 9B). *P. pentosaceus* CECT 8330 treatment significantly ameliorated the change of pathways abundance induced by DSS (Fig. 9).

### Covariance between serum cytokines, fecal SCFAs, and the gut microbiome

**Fig. 10:**
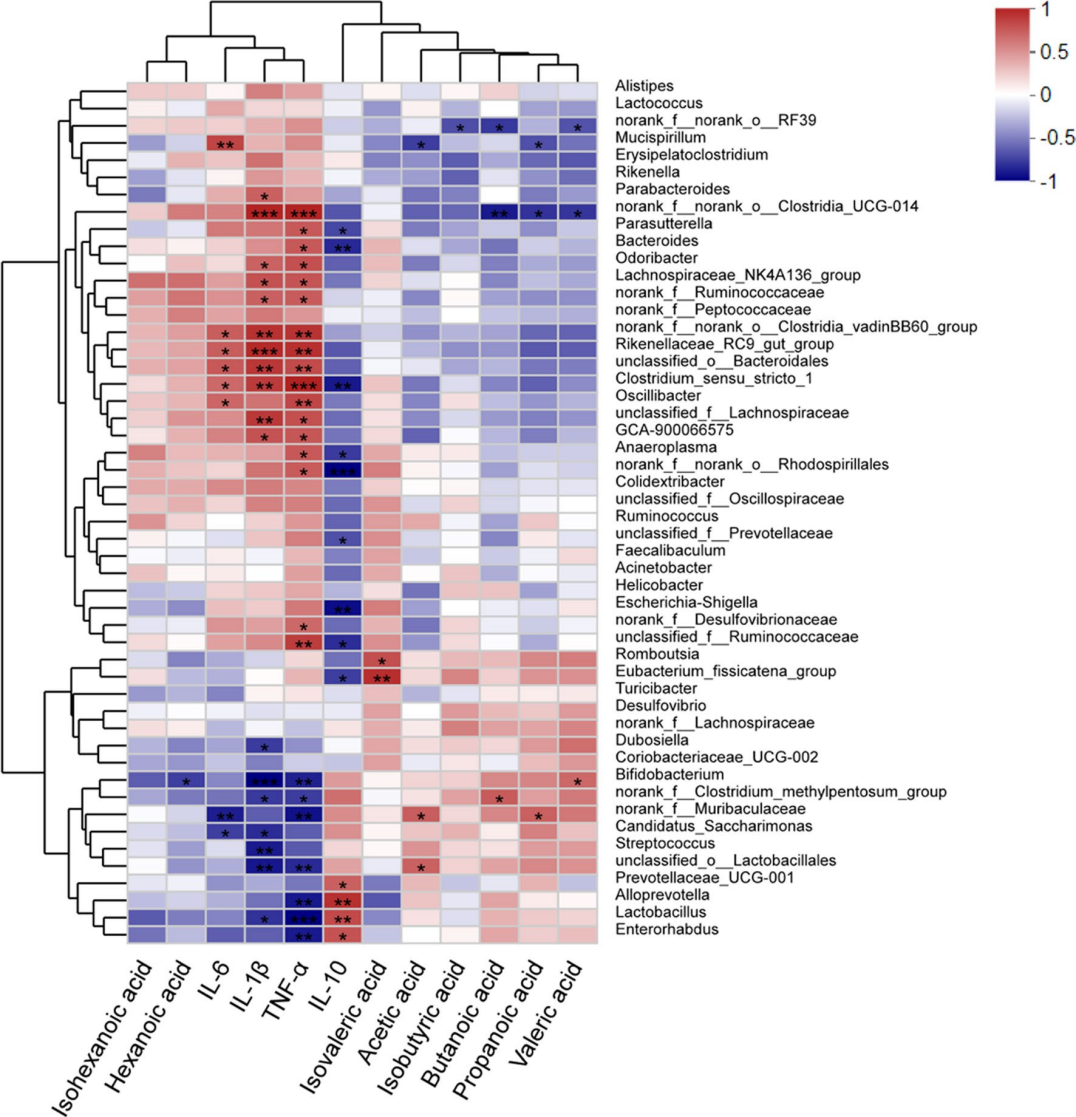
Correlations between fecal microbiome, SCFAs, and serum cytokine levels. Spearman correlation analysis were performed between top 50 abundant bacteria genera, SCFAs, and serum cytokine levels among the Control, DSS and DSS + CECT 8330 groups. Blue color represents negative correlations, and red indicates positive correlations. *P < 0.05, **P < 0.01, ***P < 0.001

To examine the relationship between members of the gut microbiota, serum cytokines, and fecal SCFAs, we performed unsupervised clustering of the top 50 abundant bacteria genera, serum cytokines and fecal SCFAs. The results revealed that the increased abundance of several genera by DSS were positively correlated with serum inflammatory cytokines (IL-6, IL-1β, and TNF-α) and negatively associated with IL-10, SCFAs, including *Lachnospiraceae_NK4A136_group*, *norank_f_norank_o_Clostridia_UCG-014*, *Clostridium_sensu_stricto_1*, *Clostridia_vadinBB60_group*, *Oscillibacter*, *GCA_900066575*, and *norank_f_Ruminococcaceae.* In contrast, several genera increased by *P. pentosaceus* CECT 8330 treatment were positively correlated with IL-10 and major SCFAs, and negatively associated with IL-6, IL-1β, and TNF-α, such as *norank_f_Muribaculaceae*, *Lactobacillus, Dubosiella*, and *Bifidobacterium* (Fig. 10)*.*

## Discussion

Changes in the gut microbiota play a critical role in the pathogenesis of IBD. Altered microbes increase susceptibility to IBD by affecting intestinal immunity and epithelial barrier function through gut microbiota-derived metabolites in genetically susceptible hosts [33]. Both quantitative and qualitative alternations of gut microbiota composition were presented in IBD patients that characterized by the decline in bacterial species and genera, and changes in the proportion of different bacteria [18]. For example, increased abundance of potentially pathogenic bacteria belonging to Proteobacteria (e.g., *E. coli* and *Klebsiella*), and reduced abundance of potentially beneficial bacteria of Firmicutes (e.g., *Faecalibacterium prausnitzii* and *Ruminococci*) were found in the majority of IBD patients [11, 34, 35]. The gut microbiota dysbiosis and dysregulated immune responses provide a strong theoretical rationale for exploring microbial-based and microbial-targeted therapies in patients with IBD. Several approaches have been used to manipulate the dysbiotic microbiota in both experimental colitis animal models and IBD patients, such as probiotics administration [21, 22].

Probiotics refer to live microorganisms that exert a beneficial effect on the health of the host when administered ingested in adequate doses [36]. To date, many probiotic strains have been investigated in IBD clinical trials. Several probiotic strains achieved a favorable outcome as mono or adjuvant therapy in IBD, especially in UC patients, such as *E. coli* Nissle 1917, LGG, *Lactobacillus reuteri* ATCC 55730, and *Bifidobacterium longum* BB536. In addition to the most studied probiotic strains that belong to *Lactobacillus* and *Bifidobacterium* genera, several other strains have been revealed to possess probiotic characteristics [37]. *P. pentosaceus* is one type of lactic acid bacteria (LAB) that belongs to the Lactobacillaceae family. As promising probiotic candidates, the functional roles of several *P. pentosaceus* strains have been investigated in recent years [38]. Many *P. pentosaceus* strains isolated from different sources were proven to be linked with the human gastrointestinal tract [38]. In animal studies, *P. pentosaceus* strains have been reported that could improve acute liver failure, obesity, constipation, fatty liver, and intestinal inflammation [39–43]. In this study, we further revealed that a *P. pentosaceus* strain isolated from healthy children, CECT 8330, protected DSS-induced experimental colitis in mice as demonstrated by inhibition of weight loss, colon length shortening, and intestinal mucous damage. Together with previous studied *P. pentosaceus* strains with anti-inflammation ability, such as *P. pentosaceus* LI05, it suggests *P. pentosaceus* may have the potential for IBD therapy [43].

Intestinal epithelial damage is the initiating event in chemically induced colitis models [27]. Our results showed that DSS significantly decreased the expression of ZO-1 and Occludin, while administration of *P. pentosaceus* CECT 8330 increased the expression of ZO-1 and Occludin that attenuated the DSS-induced epithelial damage. Inflammatory responses mediated by proinflammatory cytokines further aggravate the colitis [44]. Our data showed that the upregulated serum levels of IL-6, IL-1β, and TNF-α induced by DSS were decreased by *P. pentosaceus* CECT 8330 treatment. IL-10, an anti-inflammatory cytokine secreted by a range of immune cells, including Treg cells, plays a critical role in maintaining mucosal homeostasis [45]. It has been shown that *P. pentosaceus* CECT 8330 has the ability to induce IL-10 production in THP-1 macrophages [25]. In this study, we further showed that *P. pentosaceus* CECT 8330 increased the level of IL-10 in DSS-treated mice, which may mediated by increasing the ratio of CD4^+^CD25^+^FOXP3^+^ Treg cells in the colon.

Previous studies have suggested that the actions of probiotics are major through normalizing the altered gut microbiota [21, 22]. In this study, dysbiosis of gut microbiota by DSS treatment was determined by both *alpha* and *beta* diversity that compared with Control group. LEfSe analysis identified more than 30 taxa were differentially abundant in the Control, DSS and DSS + CECT 8330 groups, such as Phyla Firmicutes, class Bacilli, order Lactobacillales, and genera *Roseburia*, *Candidatus_Arthromitus* were enriched in Control group, order Clostridiaceae and genera *Colidextribacter, Oscillibacter* were abundant in DSS-treated mice. *P. pentosaceus* CECT 8330 treatment could significantly recover the dysregulated bacteria genera induced by DSS. Microbial marker in genus level that discriminated the three group were further identified, including high abundances of *Lactobaillus*, *Bifidobacterium*, *Candidatus_Arthromitus* in the Control group, high abundances of *Lachnospiraceae_NK4A136_group*, *Rikenellaceae_RC9_gut_group* in the DSS group, and high abundances of *unclassified_f_Prevotellaceae* in DSS + CECT 8330 group. Taken together, *P. pentosaceus* CECT 8330 treatment could partially correct the changes of gut microbiota composition induced by DSS.

Microbiota-host interactions are predominant mediated by metabolites that derived from bacterial metabolism of dietary substrates, modification of host molecules, or directly from bacteria, such as SCFAs [33, 46]. In IBD patients, the levels of SCFAs and intestinal bacteria that produce SCFAs were significantly reduced [47, 48]. Our study found decreased abundance SCFAs producing bacteria that correlated with decreased SCFAs levels in the fecal samples of DSS group as compared to the Control group, such as *norank_f_Muribaculaceae*, *Bifidobacterium*, and *Lactobacillus*. The abundance changes of SCFAs producing bacteria were most likely responsible for the decreased level of several major SCFAs, including acetic acid, propionic acid, and butyric acid. Although hexanoic acid level was slightly elevated in DSS group, *P. pentosaceus* CECT 8330 significantly reduced the hexanoic acid level in DSS + CECT 8330 group. Moreover, SCFAs exert immunomodulatory effects by increasing the number of intestinal Treg cells that maintain epithelial homeostasis [49]. We showed that *P. pentosaceus* CECT 8330 administration increased the abundances of SCFAs producing bacteria and fecal SCFAs levels. Covariance analysis further revealed that the increased abundance of several genera by DSS were positively correlated with serum inflammatory cytokines and negatively associated with IL-10, SCFAs, while several genera increased by *P. pentosaceus* CECT 8330 treatment were positively correlated with IL-10 and major SCFAs, and negatively associated with IL-6, IL-1β, and TNF-α*.*

Understanding functional interactions among microbiota within the host gut are critical to explore the pathogenesis of IBD. Studies have shown that the metabolic activities of the gut microbiota that reflected in the genes encoded into their genomes were consistently perturbed in IBD patients [9, 50, 51]. Our previous study showed that microbial function was significantly altered in the gut microbiota of pediatric CD patients that characterized by downregulation of several metabolic capacities, including KEGG pathway of replication and repair, amino acid metabolism, glycan biosynthesis and metabolism, and nucleotide metabolism [52]. Similarly, the KEGG and COG pathway analysis indicated that the dysbiosis of gut microbiota induced by DSS in mice was strongly associated with dysregulation of basic metabolic processes, such as nucleotide metabolism, glycan biosynthesis and metabolism. The declining of nucleotide metabolism could affect the metabolism of purine base by intestinal bacteria [53]. The downregulation of glycan biosynthesis and metabolism function suggested that the ability of metabolize polysaccharides into monosaccharides by gut bacteria may decrease. Furthermore, lower abundance of genes of replication and repair, translation, folding, enzyme families, infectious diseases, metabolism of terpenoids and polyketides, metabolism of other amino acids, cell growth and death, signaling molecules and interaction indicated that those pathways may be important for intestinal immune homeostasis. On the contrary, increasing abundance of genes involved in cellular processes and signaling, cell motility, transcription, signal transduction was observed in DSS group implied their roles in mediating intestinal inflammation and immune dysfunction. The KEGG and COG pathway analysis further revealed that *P. pentosaceus* CECT 8330 could partially recover the pathways altered by DSS. Given the complicated metabolic interactions occurring among bacteria and the host, more efforts are needed to uncover the role of microbial function in IBD pathogenesis in the future.

## Conclusions

In conclusion, the *P. pentosaceus* CECT 8330 administration protected the DSS-induced colitis, modulated the gut microbial composition and function, immunological profiles, and gut barrier function. Well-defined *P. pentosaceus* strains may have the potential for IBD therapy in the future.

## Supplementary Information

**Additional file 1: Fig. S1.** *P. pentosaceus* CECT 8330 protects DSS-induced colitis in male mice. (A) Schematic of animal experimental procedures (5 mice/group). (B) Changes of body weight (%). (C) Colon length shortening at day 7. (D) Representative images of the colon at day 7. (E) Disease activity index (DAI) scores. (F) Colon mucosal damage index (CMDI) scores at day 7. Significance was determined by ANOVA with Tukey’s analysis, *P < 0.05, **P < 0.01, ***P < 0.001.

**Additional file 2: Fig. S2.** *P. pentosaceus* CECT 8330 protects DSS-induced colon epithelial damage in male mice. Representative H&E- stained colon sections (magnification 100 ×) images (A, B, C, D) and histopathology score (E). Significance was determined by ANOVA with Tukey’s analysis, **P < 0.01, ***P < 0.001.

**Additional file 3: Fig. S3.** The species composition of fecal samples at phylum level among the Control, DSS and DSS + CECT 8330 groups.

**Additional file 4: Fig. S4.** The species composition of fecal samples at genus level among the Control, DSS and DSS + CECT 8330 groups.

## Data Availability

The datasets used and/or analyzed during the current study are available from the corresponding author on reasonable request.

## Abbreviations

IBD: Inflammatory bowel diseases
*P. pentosaceus*: *Pediococcus pentosaceus*
DSS: Dextran sulfate sodium
Treg: Regulatory T cells
SCFAs: Short-chain fatty acids
DAI: Disease activity index
CD: Crohn’s disease
IL: Interleukin
UC: Ulcerative colitis
TNF: Tumor necrosis factor
FMT: Fecal microbiota transplantation
LGG: *Lactobacillus rhamnosus* GG
PBS: Phosphate-buffered saline
SPF: Specific pathogen-free
CMDI: Colonic mucosa damage index
H&E: Hematoxylin and eosin
HI: Histological index
SDS: Sodium dodecyl sulfate
PMSF: Phenylmethylsulfonyl fluoride
SDS-PAGE: SDS–polyacrylamide gel electrophoresis
LAB: Lactic acid bacteria
PVDF: Polyvinylidene difluoride
LPLs: Lamina propria lymphocytes
GC–MS: Gas chromatography-mass spectrometry
OTUs: Operational taxonomic units
PCoA: Principal coordinate analysis
TJP: Tight junction proteins
