# Supplementary figures and images for: Pediococcus pentosaceus CECT 8330 protects DSS-induced colitis and regulates the intestinal microbiota and immune responses in mice
Source: J Transl Med. 2022 Jan 15;20:33. doi: 10.1186/s12967-022-03235-8 (PMC8761308; doi:10.1186/s12967-022-03235-8)

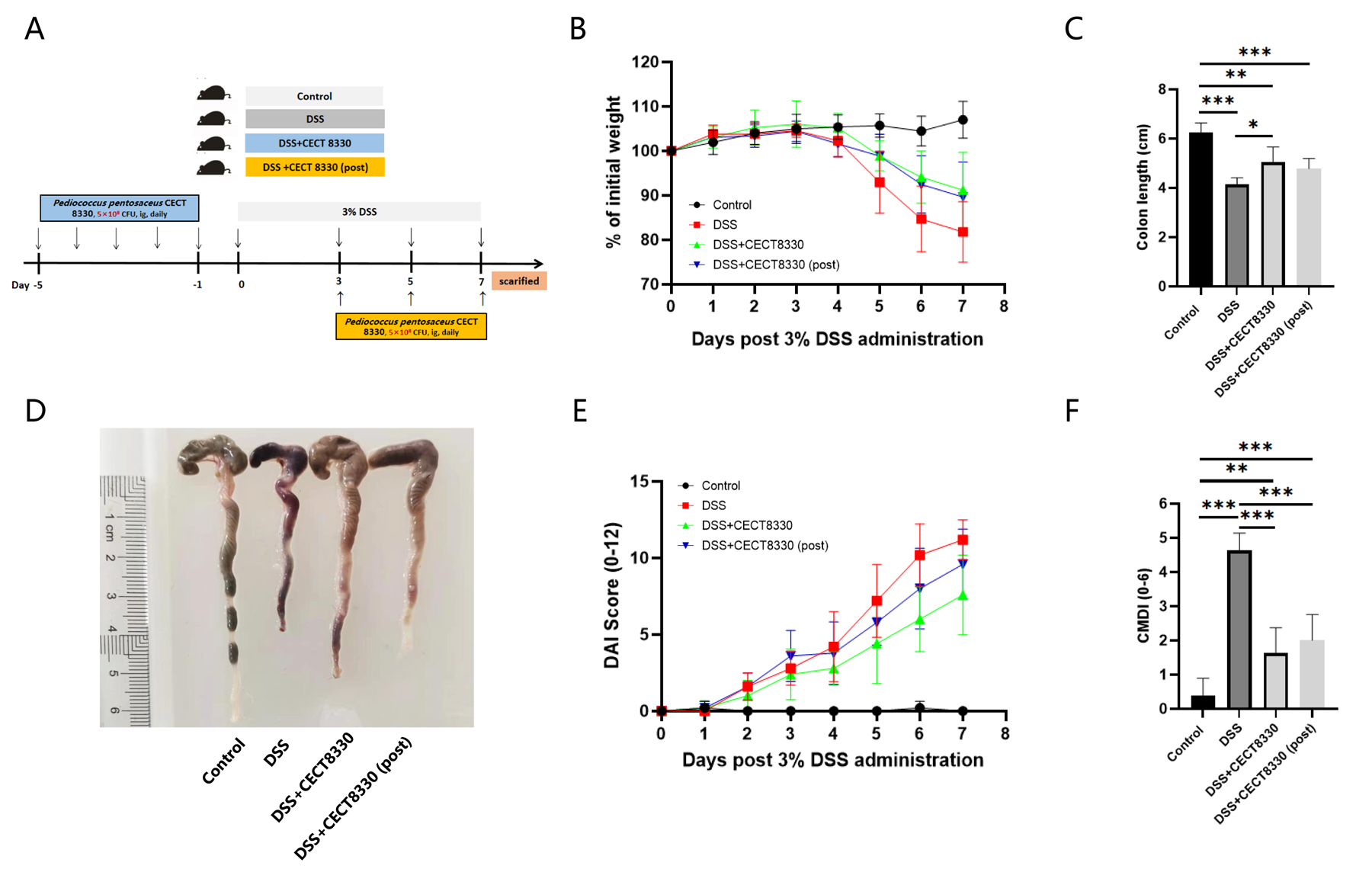

Supplement: Supplementary file 1 — Additional file 1: Fig. S1. P. pentosaceus CECT 8330 protects DSS-induced colitis in male mice. (A) Schematic of animal experimental procedures (5 mice/group). (B) Changes of body weight (%). (C) Colon length shortening at day 7. (D) Representative images of the colon at day 7. (E) Disease activity index (DAI) scores. (F) Colon mucosal damage index (CMDI) scores at day 7. Significance was determined by ANOVA with Tukey’s analysis, *P < 0.05, **P < 0.01, ***P < 0.001. [file 12967_2022_3235_MOESM1_ESM.tif]

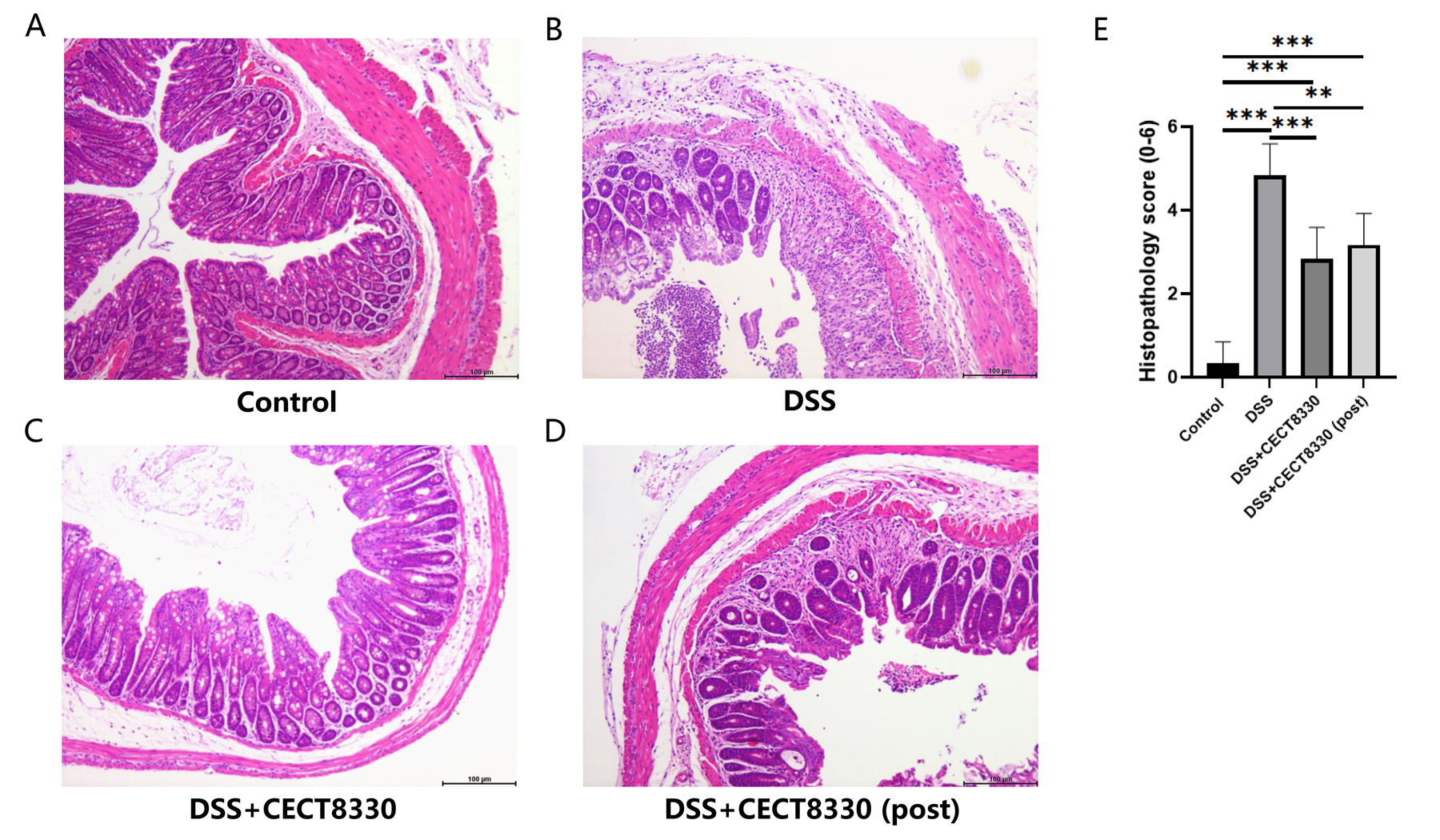

Supplement: Supplementary file 2 — Additional file 2: Fig. S2. P. pentosaceus CECT 8330 protects DSS-induced colon epithelial damage in male mice. Representative H&E- stained colon sections (magnification 100 ×) images (A, B, C, D) and histopathology score (E). Significance was determined by ANOVA with Tukey’s analysis, **P < 0.01, ***P < 0.001. [file 12967_2022_3235_MOESM2_ESM.tif]

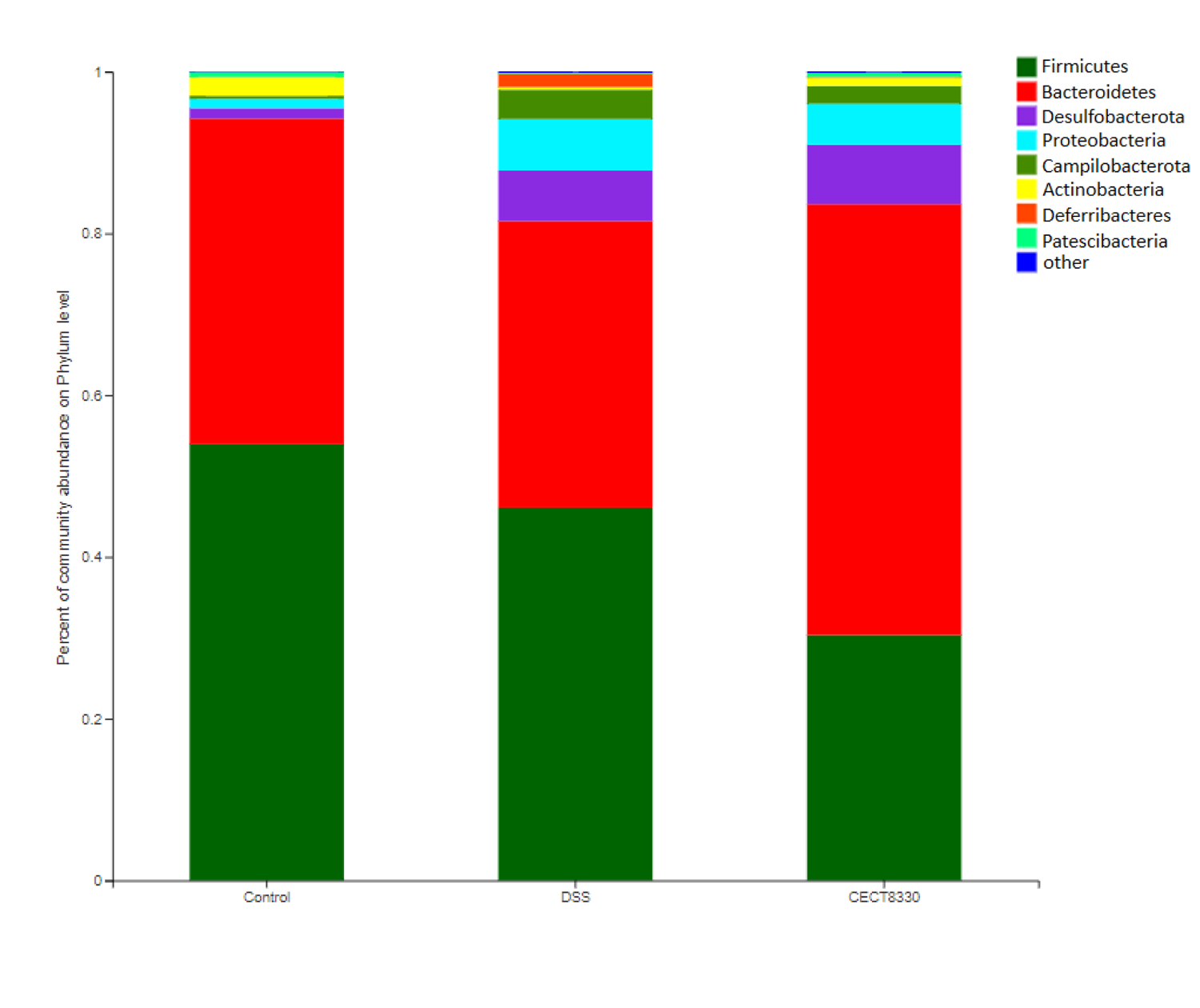

Supplement: Supplementary file 3 — Additional file 3: Fig. S3. The species composition of fecal samples at phylum level among the Control, DSS and DSS + CECT 8330 groups. [file 12967_2022_3235_MOESM3_ESM.tif]

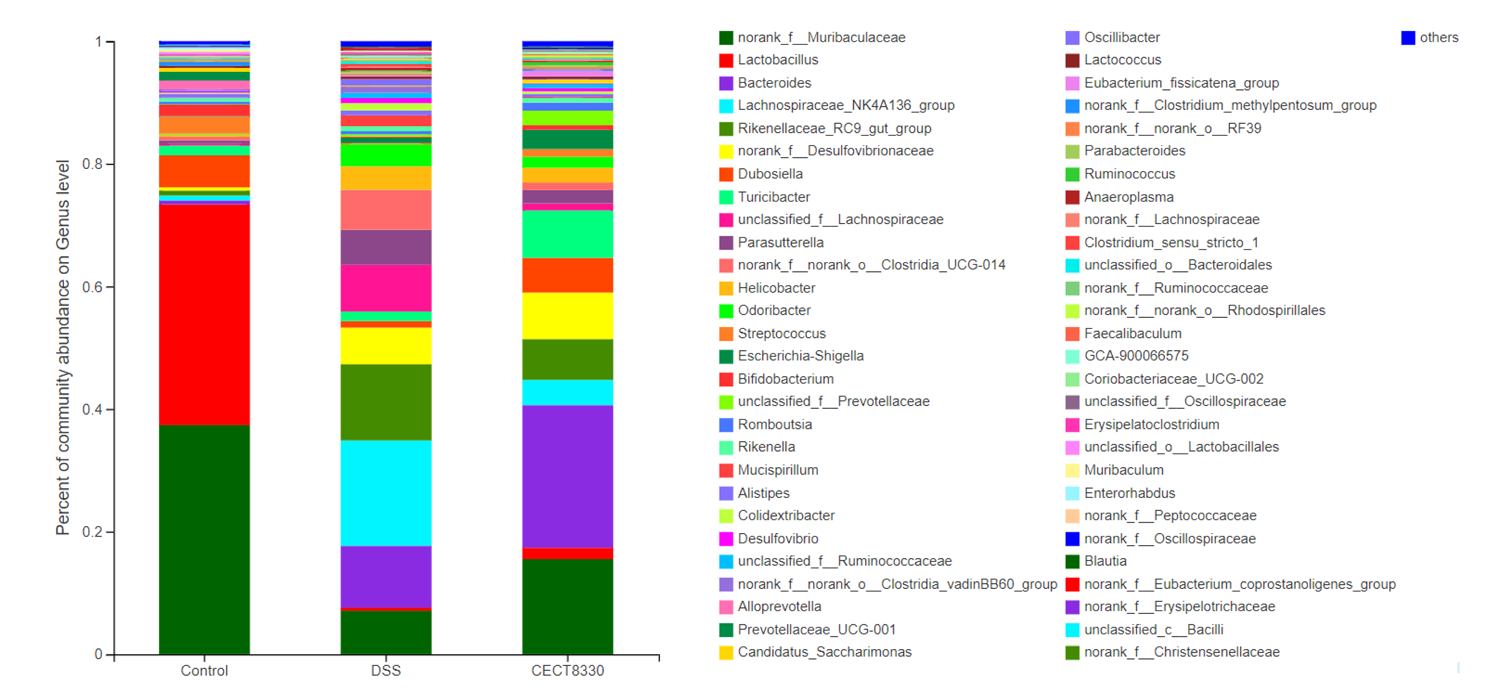

Supplement: Supplementary file 4 — Additional file 4: Fig. S4. The species composition of fecal samples at genus level among the Control, DSS and DSS + CECT 8330 groups. [file 12967_2022_3235_MOESM4_ESM.tif]
